# Editorial: Fungal toxic secondary metabolites in foods and feeds: recent sustainable analytical techniques and innovative preventative and remediation strategies for their formation and toxicity

**DOI:** 10.3389/ffunb.2024.1442327

**Published:** 2024-09-12

**Authors:** Dikabo Mogopodi, Olubukola Oluranti Babalola, Titus A. M. Msagati

**Affiliations:** ^1^ Department of Chemistry, University of Botswana, Gaborone, Botswana; ^2^ Food Security and Safety Focus Area, Faculty of Natural and Agricultural Sciences, North-West University, Mmabatho, South Africa; ^3^ Institute for Nanotechnology and Water Sustainability, College of Science Engineering and Technology, University of South Africa, Johannesburg, South Africa

**Keywords:** mycotoxins, secondary metabolites, aflatoxins, fumonisin, mycotoxin analysis

The Sustainable Development Goal to “End hunger, achieve food security and improved nutrition and promote sustainable agriculture” (SDG2) continues to be threatened by many factors, such as population increase, rising inflation, currency depreciation, disruptions in the supply chain and climate change, which have impacts on agricultural sustainability (The World Bank, 2023). Among these factors, it is also essential to recognize the role food safety plays in delaying efforts to meet SDG2 as reflected by increasing food commodity losses from contamination and increasing rate of outbreaks of foodborne diseases in many countries, especially in developing countries. The World Health Organization (WHO), for example, reports that an estimated 600 million people fall ill after eating contaminated food and 420,000 die every year, resulting in the loss of 33 million healthy life years (WHO, 2015). To reduce this burden of foodborne illnesses, multidisciplinary, urgent, and concerted efforts must be directed toward food safety.

Among food safety threats are mycotoxins, which are secondary metabolites produced by toxigenic fungi such as *Aspergillus*, *Penicillium*, and *Fusarium* species (Mogopodi et al., 2022). These species produce a variety of mycotoxins that have been the subject of much research, such as aflatoxins, fumonisin, patulin, ochratoxin A (OTA), deoxynivalenol (DON), trichothecenes: T-2 toxin, tremorgenic toxins, ergot alkaloids, and zearalenone (ZON) (Martínez-Culebras et al., 2021; Nji et al., 2022a; Fashola et al., 2023) due to their detrimental effects on human and animal health even at deficient concentrations and their economic impact. Mycotoxins are highly toxic even at very low concentrations and continue to be a major food safety problem, especially in developing countries where regulatory limits do not exist or are not adequately enforced.

The broad impacts of these toxins require integrated solutions and strategies across disciplines focusing on sensitive and selective cost-effective methods for sampling, analysis, and detection as well as innovative preventative, control, and remediation strategies. This is to ensure consumer safety and compliance with regulatory standards and focus on developing models that predict environmental factors that trigger the production of mycotoxins. This Research Topic focuses on three key areas: (i) occurrence and biological control of mycotoxins for the reduction of their impact on crops, (ii) environmental impact on mycotoxins, and (iii) method development and validation for the analysis of mycotoxins essential for the effective regulation and control of contaminants.

## Contamination of agriculture crops with mycotoxins and their control

Mycotoxins can contaminate various grain and oil crops, including maize, wheat, rice, rapeseed, soybeans, sorghum, and peanuts. Although publications indicate that 25% of crops worldwide are mycotoxin-contaminated, dating as far back as 1988, a recent study by Eskola et al. (2020) pointed out that this is likely an underestimate, with 60%–80% of crops likely mycotoxin-contaminated to some degree and 20% or more exceeding permissible food safety levels. The authors reached these estimations of mycotoxin occurrences by reviewing the literature and data of approximately 500,000 analyses from the European Food Safety Authority and a large global survey for various mycotoxins in cereals and nuts using different thresholds (Eskola et al., 2020). These staggering percentages are worrisome as crop contamination with mycotoxins cuts across the value chain, affecting farmers, traders, markets, and consumers (Smith et al., 2016). The proliferation of mycotoxins threatens global food stocks that would otherwise play a critical role in maintaining food security. It is important to come up with sustainable and green strategies to control the occurrence of various fungi and their metabolites in crops. Omotayo and Babalola reviewed the use of environmentally safe rhizosphere-associated biocontrol agents such as *Bacillus* spp., *Pseudomonas*, *Enterobacter*, and *Microbacterium oleivorans* to prevent and control *Fusarium verticillioides*—a toxicogenic fungus that produces fumonisin. They also discussed the mechanism of these microorganisms such as *Bacillus amyloliquefaciens* and *Bacillus subtilis* in reducing the level of occurrence of this fungus in the maize rhizosphere, and hence, promote crop yield.

## Environmental factors affecting mycotoxin production

Environmental conditions such as high temperature and humidity favor the growth of fungal spores, resulting in the production of mycotoxins, which are aggravated by climate change (Zingales et al., 2022; Nji et al., 2022b). These environmental impacts are also corroborated by the numerous outbreaks of acute aflatoxin exposure that have been documented in warm tropical regions, including a sizable portion of Sub-Sahara as well as Kenya and Tanzania where human fatality from aflatoxicosis has been reported (Probst et al., 2007; Kimanya et al., 2021). At elevated temperatures, an overall increase in mycotoxigenic fungal species like *Aspergillus* species has been observed (Nji et al., 2022c). The study of Ching’anda et al. demonstrated that aflatoxin-producing fungi species with differing in sclerotial morphology, specifically the L-type and S-type species of *Aspergillus* section *Flavi*, were affected differently by aflatoxins, with S-type species being highly competitive during host colonization compared to L-type species. Further, the total aflatoxins produced by these species increased at higher temperatures, with warmer temperatures favoring the growth of S-type species.

## Analytical method development and validation

The challenge of mycotoxin contamination has led to continued research growth on novel and systematic analytical techniques. Analytical method development and validation are crucial prerequisites for achieving reliable analytical data required to support mycotoxin research. These methods must be simple, effective, sensitive, and applicable to various food matrices and possibly be optimized for the analysis of multiple mycotoxins to cater to the co-occurrence of various mycotoxins. Accordingly, Mbisana et al. developed and validated a quick, easy to use, affordable, effective, robust, and safe liquid chromatography-tandem mass spectrometry (QuEChERS-LC-MS/MS) and sensitive mycotoxin analysis method encompassing the total workflow from sample preparation to quantitative analysis for the simultaneous identification and quantification of 10 mycotoxins. Within this work, the following performance characteristics of maize and sorghum were evaluated: selectivity, sensitivity, precision, and matrix effect. This method satisfied the requirements of the Commission Implementing Regulation (EU) 2021/808, Commission Regulation (EC) No. 1881/2006, and Regulation (EC) no. 401/2006.

It is not only critical to develop strategies for the analysis of mycotoxins but also to develop remediation strategies with the aim of promoting food safety. Thus, the redevelopment of green and stable adsorption materials that can remove mycotoxin is an interesting area of research. Additionally, techniques such as nanotechnology can be explored to develop such materials. It is also essential to develop affordable, non-instrumental low-cost, and convenient methods for mycotoxin analysis suitable for routine on-site screening with ease of use even for untrained personnel, which can also be for use by farmers.
